# Medical Students and Pandemic Influenza

**DOI:** 10.3201/eid1311.070279

**Published:** 2007-11

**Authors:** Benjamin Herman, Rhonda J. Rosychuk, Tracey Bailey, Robert Lake, Olive Yonge, Thomas J. Marrie

**Affiliations:** * University of Alberta, Edmonton, Alberta, Canada

**Keywords:** Pandemic influenza, medical students, knowledge, volunteering, attitudes, penalties, dispatch

## Abstract

To assess knowledge of pandemic influenza, we administered a questionnaire to all medical students at the University of Alberta; 354 (69%) of 510 students responded. Data from questionnaires such as this could help determine the role of medical students during a public health emergency.

Scientists believe we may be on the verge of the next great pandemic. Health Canada predicts that 4.5–10.6 million Canadians will become clinically ill and 11,000–58,000 will die in such an event (*1*). The current strain of influenza A (H5N1), which has been circulating in Southeast Asia among birds, with limited spread to humans, has fueled this concern (*2*,*3*). An influenza pandemic would cause a shortage of healthcare professionals due to illness and an increased demand for their services (*1*). Consequently, Canada and other countries have begun developing plans to deal with the anticipated health crisis (*1*), and medical students could play an important role.

## The Study

As part of pandemic influenza planning at the University of Alberta, researchers developed a Web-based questionnaire (*4*) comprising 42 questions to assess demographic information, risk perception of pandemic influenza (likelihood of developing and dying from the illness), general knowledge about pandemic influenza, willingness to volunteer, and suggested consequences of not volunteering during a pandemic. Response options were generally either Yes/No or a 5-point Likert-type scale (e.g., ranging from very unlikely to very likely). The final version was administered through the Internet from September 12, 2006, until October 31, 2006. The 5-point scale responses were dichotomized. Respondents with missing values were excluded from data summaries and statistical tests.

All 510 medical students at the University of Alberta were invited by email to complete the questionnaire; 354 (69%) responded. Most (62%) respondents were women, and all but 1 were reported to be in good health. A variety of past volunteer activities were reported. Although medical students obtained health information from varying sources, they had the highest degree of confidence in the information provided by physicians.

Medical students’ knowledge regarding the spread, prevention, and treatment of influenza is summarized in Table 1. Also included is the perceived likelihood of infection and outcome of such infection; 146 (41.2%) of students believed they were likely to be infected, but only 16 (4.5%) believed they would die from such an infection. Regarding volunteering during a future pandemic, most (247) believed that healthcare students have an obligation to do so (Table 2). A minority (30) agreed with penalties for heathcare students and academic staff who refused to provide services as required by the government. Healthcare and emergency workers were given the highest priority when assigning scarce resources; politicians were assigned the lowest priority.

**Table 1:** Knowledge of medical students about influenza spread, prevention, treatment, likelihood of infection, and outcome of such infection*

Survey item	No. (%)
Influenza spread through	
Close contact with infected person	287 (81.1)
Blood transfusion	88 (24.9)
Sexual contact	75 (21.2)
Cough/sneeze from infected person	338 (95.5)
Touching door knobs	263 (74.3)
Contact w/infected wild birds, chickens	182 (51.4)
Influenza prevention	
Nothing can prevent pandemic influenza	5 (1.4)
Hand washing	331 (93.5)
Cover mouth when coughing/sneezing	289 (81.6)
Vaccination	257 (72.6)
Vitamins and herbal supplements	47 (13.3)
Antiviral drugs	121 (34.2)
Antibiotics	14 (4.0)
Quarantine	241 (68.1)
Staying home/avoiding public places	253 (71.5)
Moving to area with no influenza	56 (15.8)
Wearing protective equipment in public	211 (59.6)
Eating sauerkraut	8 (2.3)
Treatment	
Nothing can treat pandemic influenza	16 (4.5)
Antibiotics	28 (7.9)
Antibacterial drugs	208 (58.8)
Vaccination	43 (12.1)
Bed rest	275 (77.7)
Fluids	280 (79.1)
Complementary medicine	19 (5.4)
Chicken soup	66 (18.6)
Likelihood of infection if pandemic influenza is in Edmonton	
Very unlikely	9 (2.5)
Likely or very likely	146 (41.2)
Likelihood (likely or very likely) of the following outcomes if you have pandemic influenza	
Won’t miss school or work	42 (11.9)
Will miss some school or work	251 (70.9)
Hospitalization but will recover	79 (22.3)
Death	16 (4.5)

*For spread, prevention, and treatment, right-hand column indicates positive response to item or statement. Multiple responses were possible within each category. For likelihood, right-hand column indicates responses for each category on a 5-point Likert-like scale ranging from “very unlikely” to “very likely.”

**Table 2:** Responses from medical students about volunteering for healthcare work during an influenza pandemic

Statement	No. (%) positive responses
Health sciences students should be encouraged to volunteer in the event of healthcare worker shortage	226 (63.8)
Retired healthcare workers should be encouraged to volunteer	185 (52.3)
Healthcare students have moral/ethical/professional obligation to volunteer	247 (69.8)
Government is justified in requiring people to work if there are insufficient volunteers	15 (43.8)
Healthcare students should be penalized for refusal to comply with government requirement	30 (8.5)
Healthcare academic staff should be penalized for refusal to comply with government requirement	76 (21.5)
Expulsion from school/termination from work	34 (9.6)
Jail time	11 (3.1)
Fine	128 (36.2)
Other	62 (17.5)
None of the above	154 (43.5)

## Conclusions

This study shows that medical students have knowledge gaps with respect to various aspects of pandemic influenza. Most medical students knew that the main route of influenza transmission is by direct contact and respiratory droplets from coughing and sneezing. However, despite no supporting medical evidence, 88 (24.9%) medical students responded that blood transfusion is a major route of transmission (*5*); 75 (21.2%) students also indicated influenza could be transmitted through sexual contact. Although direct sexual contact can transmit influenza, it is unlikely that this would be a major route of transmission.

Preventive measures are important not just during a pandemic, but at all times to decrease the risk of transmission of bacterial and viral illnesses. Most medical students (93.5% and 81.6%, respectively) correctly identified hand washing and cough etiquette, respectively, as effective preventive measures (it is important to note, however, that there is a difference between students knowing and complying). Social distancing can help reduce the peak incidence of an epidemic and spread it over several weeks instead of a few, thus avoiding a healthcare surge (*6*). Household quarantine is also effective at reducing attack rates in the community but only if the compliance rate is high (*7*). Knowledge regarding treatment of pandemic influenza is difficult to analyze because determining what measures would be effective is complex. Given the time lag between identification of the pandemic strain and the time needed to produce a vaccine, any known treatment strategy would not be effective unless it could be applied to the strain that is causing the pandemic.

Although it is encouraging that most students believe healthcare students have an obligation to volunteer during a pandemic, a dichotomy exists when it comes to setting a policy for actual deployment. One argument is that medical students should be encouraged to volunteer during a crisis because this will provide valuable medical training at a time when a great deal of the workforce—including physicians and nurses—will be ill and unable to work. During the 1918 influenza pandemic, some medical students in the United States graduated early from medical school and had expedited medical board examinations to increase the number of medical personnel available to provide services (*8*). Conversely, some may argue that placing the next generation of doctors on the front lines as volunteers will put them at increased risk for contracting illness. It is possible in this situation that the numbers of future physicians could decrease and adversely affect recovery efforts after a pandemic. During the severe acute respiratory syndrome crisis in Toronto, medical students were removed from the wards (D. Low, pers. comm.) (*9*). Although this was done with good intentions and safety in mind, students experienced a great deal of frustration regarding the suspension of their education and research activities (*9*).

Other recent events may offer insight into the desirability of using medical students as volunteers. Anecdotes from the terrorist attacks in New York City on September 11, 2001, relate how medical students helped in various capacities immediately following the tragedy (*10*). However, no official school or government mandate was issued forcing these persons to volunteer. In contrast, after the devastation of Hurricane Rita, college leaders at Texas A&M University College of Medicine made a decision to cancel classes and provided orientation sessions for students so that they could begin volunteering as soon as possible (*11*). Volunteer tasks in this situation were limited, and many voiced frustration that their relatively limited medical skills greatly restricted their ability to contribute. In a more positive vein, students also claimed that “the experience made them even more driven to become the best doctors they can” (*11*).

Our study was limited in that only 69% of the medical students at 1 medical school in Canada responded. However, the results do show that planning and debate should begin before a pandemic occurs and that expectations of the role of medical students must be explicit (*12*). A government must consider the virulence and characteristics of the illness as well as the number of volunteers needed before deciding on an appropriate course of action. Ultimately, it remains to be seen what the climate of the next pandemic will be.
